# Current View on PPAR-α and Its Relation to Neurosteroids in Alzheimer’s Disease and Other Neuropsychiatric Disorders: Promising Targets in a Therapeutic Strategy

**DOI:** 10.3390/ijms25137106

**Published:** 2024-06-28

**Authors:** Sylwia Żulińska, Anna K. Strosznajder, Joanna B. Strosznajder

**Affiliations:** 1 Department of Cellular Signaling, Mossakowski Medical Research Institute, Polish Academy of Sciences, 5 Pawińskiego St., 02-106 Warsaw, Poland; swojtowicz@imdik.pan.pl; 2 Department of Psychiatry, Medical University of Warsaw, Nowowiejska St. 27, 00-665 Warsaw, Poland; anna.strosznajder@gmail.com

**Keywords:** Alzheimer’s disease, neurosteroids, neurodegenerative disorders, neuropsychiatric disorders

## Abstract

Peroxisome proliferator-activated receptors (PPARs) may play an important role in the pathomechanism/pathogenesis of Alzheimer’s disease (AD) and several other neurological/neuropsychiatric disorders. AD leads to progressive alterations in the redox state, ion homeostasis, lipids, and protein metabolism. Significant alterations in molecular processes and the functioning of several signaling pathways result in the degeneration and death of synapses and neuronal cells, leading to the most severe dementia. Peroxisome proliferator-activated receptor alpha (PPAR-α) is among the processes affected by AD; it regulates the transcription of genes related to the metabolism of cholesterol, fatty acids, other lipids and neurotransmission, mitochondria biogenesis, and function. PPAR-α is involved in the cholesterol transport to mitochondria, the substrate for neurosteroid biosynthesis. PPAR-α-coding enzymes, such as sulfotransferases, which are responsible for neurosteroid sulfation. The relation between PPAR-α and cholesterol/neurosteroids may have a significant impact on the course and progression of neurodegeneration/neuroprotection processes. Unfortunately, despite many years of intensive studies, the pathogenesis of AD is unknown and therapy for AD and other neurodegenerative diseases is symptomatic, presenting a significant goal and challenge today. This review presents recent achievements in therapeutic approaches for AD, which are targeting PPAR-α and its relation to cholesterol and neurosteroids in AD and neuropsychiatric disorders.

## 1. Introduction

**Figure 1:**
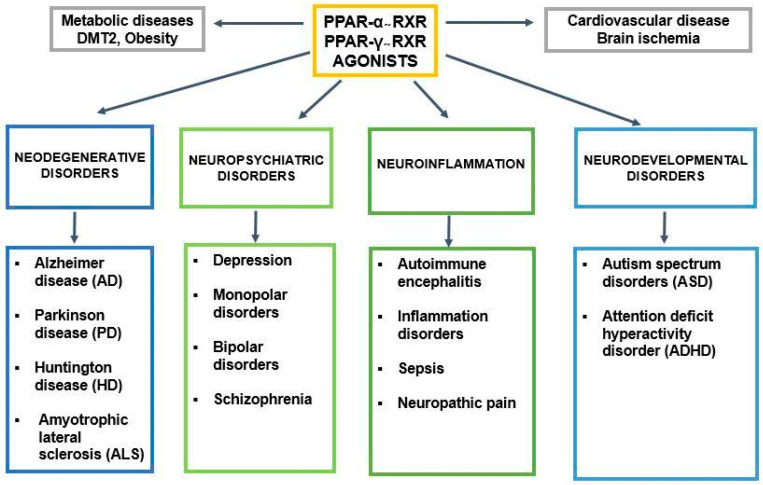
PPAR-α and its engagement in metabolic, cardiovascular, and neurological/neuropsychiatric disorders.

Alzheimer’s disease (AD) and several other neuropsychiatric disorders are among the most significant unsolved medical and social problems. Despite intensive studies and significant progress in genetics and molecular biology, the pathomechanism/pathogenesis of AD and most other neurodegenerative/neuropsychiatric diseases such as Parkinson’s disease (PD), Huntington’s disease (HD), and schizophrenia has not been fully elucidated, and therapeutic approaches are still not effective. Several million people around the world are waiting for novel or repurposing drugs [1,2,3] that will be able to retard or ameliorate neurodegeneration. Unfortunately, along with major progress in several fields of science and technology, an increase in psychiatric disorders has been noticed [4]. In the last decades, a significant rise in psychosis, depression, schizophrenia, and several neurodevelopmental diseases, including autism spectrum disorder (ASD) and attention deficit hyperactivity disorder (ADHD), has been reported [5,6]. Statistical analysis from the WHO reveals a 13% increase in mental health disorders over the past 10 years. In AD and other neuropsychiatric and neurodevelopmental diseases mentioned above, neuroinflammation could play an important role. It should be highlighted that in all these diseases, alterations in peroxisome proliferator-activated receptor (PPAR-α, PPAR-γ) signaling may play a crucial pathogenic role (Figure 1).

**Figure 2:**
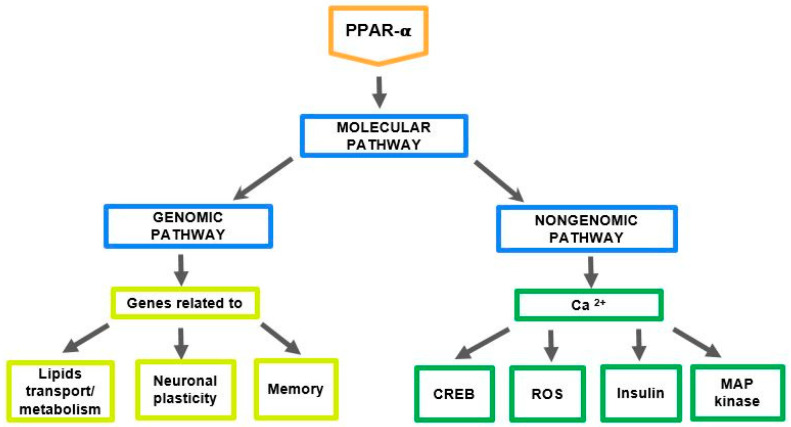
PPAR-α and the molecular mechanism of its action through genomic and nongenomic pathways. Ca^2+^—Calcium ions, CREB—cAMP-response element-binding protein, MAP kinase—mitogen-activated protein kinase, ROS—reactive oxygen species, according to Roy et al. [14,15] and Bougarne et al. [16].

For the first time, the role of PPARs was described in cardiovascular disorders and metabolic diseases, such as dyslipidemia and type 2 diabetes (DMt2). These diseases and obesity also affect cognition and memory [7,8]. Agonists of PPAR-α exert several positive effects on these disorders [9,10,11,12,13]. PPARs regulate cell metabolism through genomic and non-genomic pathways (Figure 2).

PPAR-α is a potent transcription factor that regulates genes related to fatty acids (FA) transport and oxidation, cholesterol transport/sulfation, and triglyceride (TG) metabolism. Moreover, PPAR-α regulates the transcription of various neuronal plasticity and memory-associated genes encoding the NMDA receptor subunits (NR2A, NR2B), AMPA receptor subunit (GluR1), and activity-regulated cytoskeleton-associated protein (Arc). Additionally, PPAR-α exerts an effect through the nongenomic pathway by regulating intracellular Ca^2+^ concentration (Ca^2+^i). PPAR-α via rapid enhancement of Ca^2+^i significantly influences the level of ROS production, mitogen-activated protein kinases (MAPKs), the insulin level, and the expression of cAMP-response element binding (CREB), which is important in the formation of memory [14,15]. Recent studies highlight the crucial role of the immediate early gene Arc/Arg 3.1 in synaptic plasticity and memory consolidation. Arc is activated by natural stimuli and memory-related behavioral paradigms. The Arc/Arg3.1 protein is present in the postsynaptic density of glutamatergic neurons [17,18,19].

**Figure 3:**
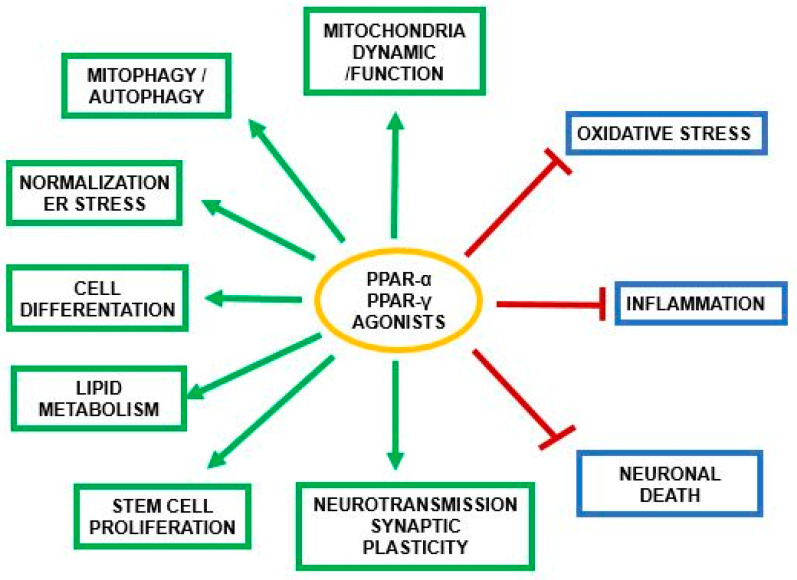
PPAR-α and its role in the activation or inhibition of crucial processes engaged in the survival and death of brain cells.

PPAR-α and other members of the PPAR family have been previously described in several original and review articles [10,11,12,13,20]. In this review, we would like to highlight the basic role of each PPAR receptor in the central nervous system (CNS). All members of these receptors are potent transcription factors; PPAR-α was discovered and characterized as the first. It is widely present in CNS and is engaged in the regulation of mitochondrial fatty acid and cholesterol metabolism. Moreover, this receptor plays an important role in mitochondrial function, energy metabolism, biogenesis, and oxidative stress. PPAR-α plays a crucial role in neurotransmission processes in glutamatergic, cholinergic, and dopaminergic systems, in synaptic plasticity, and in the regulation of mitophagy/autophagy and neuronal death pathways. PPAR-α and PPAR-γ have significant effects on cell differentiation and inflammation [21,22] (Figure 3).

PPAR-β/δ is mostly present in CNS, similar to PPAR-α, and is involved in cell differentiation and myelination [12,13]. PPAR-γ is distributed in most of the cells in the human body. The agonists of PPAR-α and PPAR-γ participate in brain signaling, mitochondrial metabolism, and mitophagy. They also take part in the normalization of endoplasmic reticulum (ER) stress, synaptic plasticity, and other processes indicated in Figure 3. Additionally, both of these receptors could play a significant role in lipid and fatty acid metabolism. Moreover, PPAR-α is engaged in cholesterol metabolism and the biosynthesis of neurosteroids.

## 2. The Role of PPAR-α in the Regulation of Genes Related to Cholesterol and Neurosteroids

**Figure 4:**
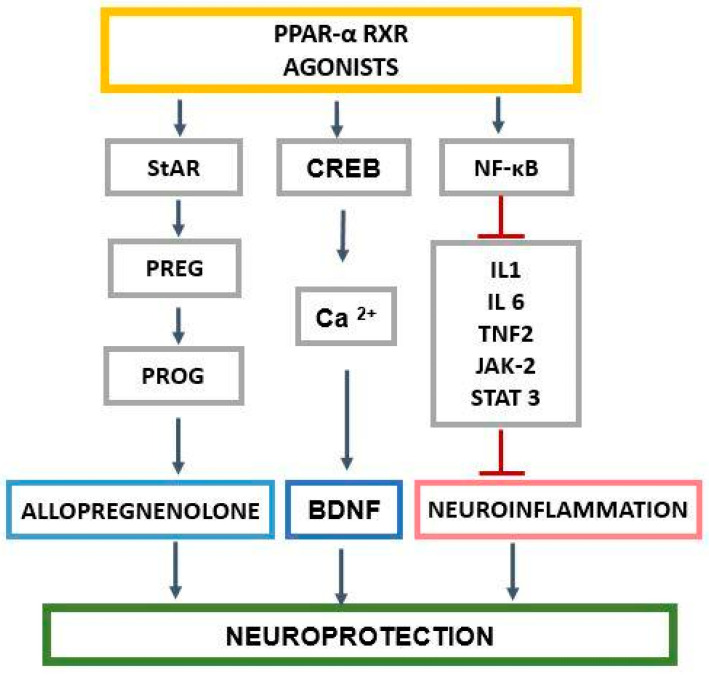
PPAR-α and its role in neuroprotection, through neuroactive steroids, the CREB/Ca/BDNF pathway, and NF-κB signaling. StAR—steroidogenic acute regulatory protein, CREB—cAMP-response element binding protein, PREG—pregnenolone, PROG—progesterone, BDNF—brain-derived neurotrophic factor, NF-κB—nuclear factor kappa-light-chain-enhancer of activated B cells, IL-1—interleukin 1, IL-6—interleukin 6, TNF2—tumor necrosis factor-alpha promoter variant 2, JAK-2—Janus kinase 2, STAT3—signal transducer and activator of transcription 3, Ca^2+^—calcium ions. According to Pinna [24] and Roy et al. [14].

The relationship between PPAR-α and neurosteroids in CNS could be very important for the pathomechanism and treatment strategies of neuropsychiatric disorders as described previously by Nisbett and Pinna [23]. The activation of PPAR-α may exert neuroprotection through the regulation of genes involved in several signaling pathways and those encoding proteins engaged in cholesterol transport into mitochondria and its metabolism to neurosteroids. Cholesterol is the substrate for neurosteroids, and its level may influence the synthesis of progesterone, allopregnanolone, and other compounds (Figure 4).

Moreover, PPAR-α may exert an effect through the CREB-mediated pathway and BDNF biosynthesis or by NF-κB-mediated inhibition of neuroinflammation. PPAR-α may also influence neuroinflammation processes, depression, and cognition/memory functions indirectly through neurosteroids (neuroactive steroids).

PPAR-α and neurosteroids play an important role in many processes, including cognition, memory, and emotions. Recently, Ratner et al. [25] explained how neurosteroids might affect memory and memory deficits. The regulatory roles of endogenous and synthetic neurosteroids on gamma-aminobutyric acid (GABAA) and N-methyl-D-aspartate (NMDA) receptors function were evaluated. Neuroactive steroids exert effects through GABAA, GABAB, and NMDA receptors and by 5-hydroxytryptamine type 3 (5-HT3) and sigma 1 receptors [26,27]. The involvement of neurosteroids in the etiology and treatment of learning and memory disturbances was previously reported. Additionally, the role of neurosteroids in neuropsychiatric diseases, such as schizophrenia, depression, and anxiety, has been analyzed over the last 25 years by several research groups [25,28,29,30,31,32,33,34].

**Figure 5:**
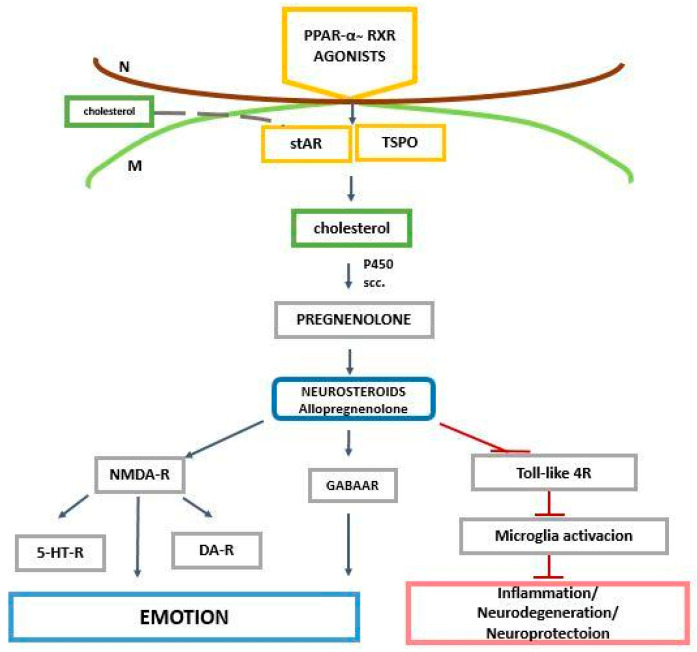
Relation between PPAR-α and cholesterol/neurosteroids and their effect on GABAA, NMDA, and toll-like receptors in the regulation of emotions and neurodegeneration/neuroprotection. N—nucleus, M—mitochondria, NMDA—N-methyl-D-aspartate, StAR—steroidogenic acute regulatory protein, TSPO—translocator protein, NMDA-R—N-methyl-D-aspartate receptor, 5-HT-R—5-hydroxytryptamine receptor, DA-R—dopamine receptor(s), gamma-aminobutyric acid type A receptor—GABAAR, P450scc—cytochrome P450 side-chain cleavage, TPSO—translocator protein.

The question arises of how PPAR-α is related to neurosteroids. It is important to highlight that PPAR-α plays a crucial role in the transcription of two genes engaged in the transport of cholesterol into mitochondria. One of these genes is responsible for encoding the steroidogenic acute regulatory protein (StAR, STARD1) and another one encodes the translocator protein (TSPO) (Figure 5).

**Figure 6:**
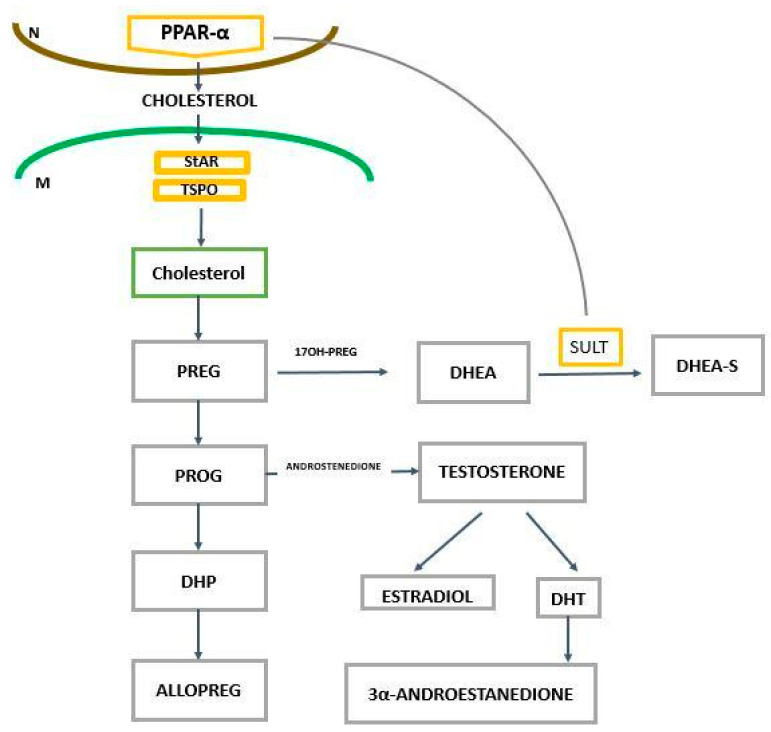
Effects of PPAR-α on neurosteroids and their sulfation. N—nucleus, M—mitochondria, StAR—steroidogenic acute regulatory protein, TPSO—translocator protein, PREG—pregnenolone, PROG—progesterone, ALLOPREG—allopregnanolone, DHP—5α-dihydroprogesterone, DHT—dihydrotestosterone, DHEA—dehydroepiandrosterone, DHEA-S—dehydroepiandrosterone sulfate, SULT—sulfotransferase.

In AD, the disruption of intracellular cholesterol was reported and the accumulation of cholesterol in mitochondria may affect antioxidative processes and enhance Aβ toxicity. Moreover, the alteration of cholesterol homeostasis may evoke endoplasmic reticulum stress, which may lead to the inhibition of acid ceramidase, the accumulation of ceramides, and cell death as described by Torres et al. [35]. These authors suggest that StAR/STARD1 could be a promising target in the therapy of AD. However, it seems that the PPAR-α ligands, which regulate the transcription of genes related to cholesterol transport into mitochondria and several other genes, could be more effective and safer and could concomitantly exert a positive effect on lipid metabolism and neuroinflammation [16]. The transport of cholesterol through the outer mitochondria membrane (OMM) to the inner mitochondria membrane (IMM) occurs via the cytochrome P450 side-chain cleavage enzyme (P450scc). This protein is encoded by the *CYP11A1* gene, and is the first and rate-limiting step in steroid biosynthesis, involved in the metabolism of cholesterol into pregnanolone, and is the precursor of several other steroid hormones [36,37] (Figure 6).

Neuroactive steroids are natural derivatives of progesterone and are positive allosteric modulators of GABA receptors. Researchers have suggested that the organization and regulation of GABA receptor subunits may affect the profile of action. It has also been indicated that inflammatory pathways are targets for neurosteroids and could be responsible for the beneficial actions of PPAR-α. However, neuroinflammation may influence neurosteroid synthesis and the profile of their actions.

Recently it was described that the metabolism of mitochondria cholesterol play significant role in redox biology and in AD pathology Goicoechea et al. [37]. These authors highlighted the importance of mechanisms involved in the regulation of cholesterol levels and their metabolism in mitochondria in physiology and pathology. Researchers noticed that the accumulation of cholesterol in mitochondria above the physiological level may affect the assembly of respiratory supercomplexes and may activate oxidative stress. Enhanced levels of cholesterol could exert a negative impact on the antioxidative defense through the glutathione redox cycle.

**Figure 7:**
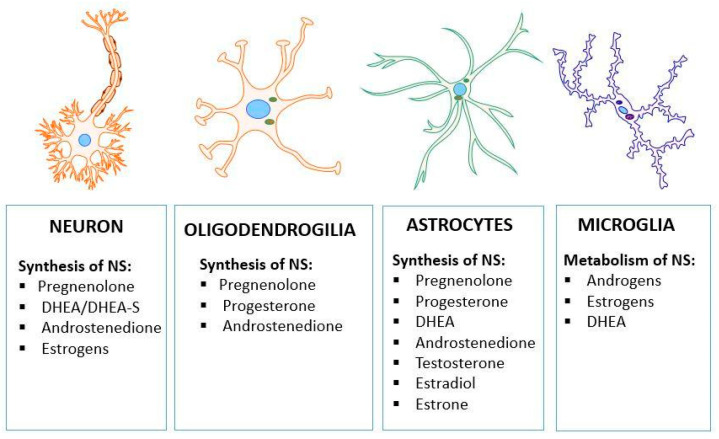
Biosynthesis and metabolism of neurosteroids (NSs) in different brain cells, according to Zwain and Yen [45], Gago et al. [46], and Alexaki et al. [47].

The mitochondrial pool of cholesterol is under precise control and it seems that PPAR-α may play a crucial role in the regulation of cholesterol homeostasis. Mitochondria cholesterol represents about 2–4% of the total pool of cholesterol in the cells and is indispensable for the synthesis of steroids, oxysterols, and hepatic bile acids [37,38]. For many years, it has been suggested that a close association between cholesterol levels/metabolism and AD pathology exists [39,40,41,42,43]. Recently, Testa et al. [44] noted that 24-hydroxycholesterol (24-OHC) induces Tau proteasome-dependent degradation via the Srt1/PGC-1α/NRF2 pathway. Through this mechanism, 24-OHC may protect the brain against the accumulation of the hyperphosphorylated Tau protein and its neurotoxic effect. Researchers have also suggested that cholesterol—depending on the concentration and metabolism—could play a different role but its homeostasis is crucial for the functioning of the cells. Up until now, the role of cholesterol in AD and other neuropsychiatric disorders has not been fully elucidated. Cholesterol metabolism depends on many factors, including several enzymes, cells, and parts of the brain. In neurons, the conversion of cholesterol to pregnenolone (PREG), the precursor of steroid hormones, is catalyzed by cytochrome P450 side-chain cleavage (P450scc). In neurons, pregnenolone is synthesized as well as DHEA/DHEA-S, androstenedione, and estrogens [45] (Figure 7).

Moreover, other enzymes, including 5α–reductase and 3α-hydroxysteroid dehydrogenase, are crucial in the synthesis of allopregnanolone (ALLO) and tetrahydrodeoxycorticosterone (THDOC). These neurosteroids can be synthesized in the hippocampus, brain cortex, thalamus, and olfactory bulb. The sulfation and desulfation alter the neurosteroid compounds and modify their properties. PPAR-α plays a key role in the regulation of gene expression coding sulfotransferase (SULT) and UDP-glucuronosyltransferase [48]. Pregnenolone and allopregnanolone are present in the brain in greater concentrations compared to blood and both pools significantly affect brain function, inflammation, and autophagy [49,50]. It is widely accepted that inflammation contributes to the pathogenesis of AD and other neuropsychiatric disorders; moreover, inflammation plays a crucial role in neurodevelopmental disorders and it may evoke these pathologies.

Based on preclinical studies, researchers have postulated that the alteration of brain cholesterol biosynthesis could be responsible for the changes in cognitive function in AD [51,52,53,54]. However, this subject is still under discussion [44,53]. The blood–brain barrier is not permeable to cholesterol/lipoproteins, which means that brain cholesterol must be synthesized de novo in the brain. This biosynthesis is especially active during myelination. Cholesterol content in the brain is higher 10 times as compared to other organs and tissues and consists of about 25% of the total body cholesterol. This lipid is an essential compound of all cell membranes, as it forms the lipid rafts together with sphingolipids. These lipid rafts create an appropriate milieu for most of the different types of neurotransmitter receptors, ion channels, and amyloid beta precursor proteins (APPs), whose functions are not yet fully explained. However, it is well known that APP-altered metabolism in AD is responsible for the higher liberation of amyloid β, which may play a crucial role in the pathogenesis/pathomechanism of the familiar/genetic form of early-onset AD (EOAD). In sporadic late-onset AD (LOAD), which affects more than 90% of AD patients, pathogenesis/pathomechanism is unknown and therapy is unsuccessful. However, in this form of AD, altered metabolism of APP and the toxicity of liberated excess amyloid beta also occur and play an essential but not crucial role. Sáez-Orellana et al. [55] indicated an important correlation between the PPAR-α function and full-length APP expression in LOAD and EOAD. The authors reported that PPAR-α expression and activation were inversely related to APP expression in LOAD and in EOAD but not in the control human brain. PPAR-α deficiency eliminates APP–mediated control of synaptic activity, demonstrating the key role of PPAR-α in this process. Moreover, the alteration of APP metabolism impairs lipid synthesis and synaptic activity, while the activation of PPAR-α improves synaptic plasticity through its effect on lipid metabolism [55]. It could be suggested that PPAR-α may also exert its protective effect on synaptic function through the regulation of cholesterol transport in brain mitochondria and the biosynthesis of neurosteroids.

Neuroactive steroids are synthesized de novo in the brain from cholesterol in neurons, oligodendroglia, and astrocytes, and metabolized in microglia, as demonstrated in Figure 7. Neurosteroids are sulfated by sulfotransferases and transcription is regulated by PPAR-α [34,56,57]. Dehydroepiandrosterone (DHEA) and its sulfated form, DHEA-S, are the most abundant forms of steroids. More than 90% of circulating DHEA is in the sulfated form (as DHEA-S), which powerfully binds to albumins. DHEA and progesterone are precursors for the major sex steroids (estrogen, progesterone, and testosterone). These sex compounds are mainly synthesized in adrenal glands but some are also synthesized in the brain. DHEA and its metabolites decrease significantly during aging and the DHEA-S level declines by about 80% by age 70 compared to adult levels [58]. A lower concentration of DHEA-S in plasma and reduced activity of sulfotransferases are considered as risk factors for AD. A lower level of neurosteroids in the brain during aging and in AD could be responsible for the reduced steroid neuroprotective effect. Consequently, the brain is more sensitive to neurotoxins, such as cortisol, and other damaging factors, such as Aβ oligomers. However, about 50 years ago, it was found that sex sterols synthesized in gonads and other periphery organs can cross the blood–brain barrier (BBB) and may exert their effect in the brain, but the level of these compounds significantly decreases during aging.

Ongoing research with neuroactive steroids will elucidate the effects of these compounds on the molecular processes in the brain. There is high hope that neuroactive steroids will improve the treatment of depression [59]. Recently developed, rapidly acting antidepressants are promising in the therapy of depression and psychosis, which often appear during AD or PD progression. In 2019, the FDA approved brexanolone, a type of neurosteroid, which exerts a positive effect on postpartum depression (PPD) as well as in the treatment of major depressive disorders (MDDs). The novel rapid-acting neurosteroids were suggested to be able to significantly change the therapeutic strategy of depression and schizophrenia. After many years of treatments with several classic anti-depressants, this FDA-approved allopregnanolone (brexanolone) and antidepressant esketamine (as nasal spray) for PPD and resistant depression, respectively, are expected to significantly improve the treatment of depression [49,60]. Moreover, the FDA also approved zuranolone, an oral allopregnanolone analog, for both of these types of depression. These compounds act on GABA and NMDA receptors and also have anti-inflammatory agents [61]. It is postulated that the new era for the treatment of neuropsychiatric disorders has just started with the introduction of these rapid-acting psychotropic drugs, including supplementation with dehydroepiandrosterone (DHEA), as proposed by Baulieu et al. [36].

Many years ago, Brown et al. [62] and Zwain and Yen [45] described neurosteroid biosynthesis in cell lines from the brain and regulation of DHEA synthesis via oxidative stress and β-amyloid peptides. Nowadays, neurosteroids/neuroactive steroids (NAS), neurosteroid enantiomers, and sulfated neurosteroid enantiomers are expected to be very efficient in the treatment of several neuropsychiatric/neurodegenerative disorders [25,59,63,64]. Unfortunately, recently several side effects were reported; these drugs should be carefully applied with very restricted, appropriate doses.

Previously, Grimm et al. [65] analyzed the effects of seven structurally diverse neurosteroids, which may have implications for age-related neurodegenerative disorders. Several neurosteroids, such as progesterone, estradiol, estrone, testosterone, DHEA, and allopregnanolone, exert positive effects on the redox state by activating antioxidative defense and mitochondria function. These steroids improve mitochondrial respiration and the synthesis of ATP in an age-dependent manner and can positively influence the membrane’s potential. It seems that neurosteroids—acting through their specific receptors—could be involved in neuroprotective events, which occur concomitantly with degenerative processes during aging or progressive neurodegenerative diseases, such as AD or PD. Allopregnanolone has been shown to exert a neuroprotective effect through several molecular pathways, including the GABA receptor, CREB, and Ca-regulated processes. It significantly activates neurogenesis, a proliferation of neuronal progenitor cells (NPCs). Moreover, it promotes the clearance of cholesterol and maintains its homeostasis and Aβ peptide homeostasis. Allopregnanolone and testosterone exert positive effects by modulating the GABAA receptor, by reducing glutamate release, IL1β, and TNF expression. It seems that through these pathways, neurosteroids may also exert an effect on neuropathic pain. Moreover, these neurosteroids act through other mechanisms, including the activation of factors related to peripheral myelination, as indicated in preclinical studies involving animal models [66,67]. Additionally, neurosteroids improve mitochondrial activity and functions [65,68]. An enigmatic relationship between estrogen and mitochondria in AD was described by Grimm et al. [69]. It has been known for a long time that mitochondria are highly vulnerable to Aβ and tau toxicity and it was suggested that mitochondrial dysfunction could be an early event during aging and AD/PD pathology. It was demonstrated that the synthesis of neurosteroids decreased during aging. The lower concentrations of DHEA and other neuroactive steroids exert significant effects on most intracellular signaling pathways and autophagy, affecting memory and learning ability.

The previous study indicated a close link between PPAR-α, the neurosteroidogenic pathway, and the GABA receptor. This pathway mediates signaling in several neuropsychiatric disorders, including post-traumatic stress disorder (PTSD), depression, schizophrenia, substance use disorders, drug addiction, and several neurodegenerative disorders, such as AD and PD [23,70,71,72,73]. Recently, Covey et al. [63] highlighted that there has been significant interest in the last few years in developing neurosteroid enantiomers as novel promising compounds for the treatment of AD. The lower concentrations of DHEA-S and other neuroactive steroids have significant effects on most intracellular signaling pathways, inflammation, and autophagy. It is expected that—in an appropriate dose—neurosteroids will be able to improve the treatment of neurodegenerative and neuroinflammatory disorders. In most neurodegenerative diseases and neuropsychiatric disorders, neuroinflammation plays a crucial role [16,74]. Neuroinflammation may be neuroprotective, however, it may also lead to neurodegeneration or even evoke neurodegenerative disorders. In the last few years, several pieces of data, which have been obtained from positron emission tomography (PET) imaging in humans, have enhanced our understanding of the association between neuroimmune responses and pathology in AD and other brain disorders. Positive results have recently been obtained with second-generation radioligands, markers of immune response in PET imaging of neuroinflammation. PET imaging could play a role in drug discovery and the evaluation of drug effects, but PET studies have some limitations [75].

The PPAR-α ligand fenofibrate (FF) has a positive effect on neuroinflammation processes activated in neurodegenerative/neurodevelopmental disorders. Prenatal fenofibrate administration attenuated the detrimental effect evoked by maternal inflammation activation (MIA) on the schizophrenia-like behavior phenotype and dopamine transmission in male offspring. Polyunsaturated fatty acids omega- 3 PUFA, which are PPAR-α ligands, prevent the transition to psychosis in individuals at ultra-high risk for psychosis. PPAR-α activation inhibits inflammation by reducing oxidative stress, liberating cytokines such as IL1, IL6, and TNF-α, and decreasing pro-inflammatory enzymes [76]. The association between PPAR-α and neurosteroids is crucial in the progression of several neurodegenerative and neuropsychiatric disorders [24]. Neurosteroids could be very potent regulators of neuroinflammation. On the other hand, neuroinflammation may affect the synthesis of neurosteroids [49]. It is very important to highlight the sex differences in responses to the PPAR-α ligand fenofibrate, but no information on the actions of other PPAR ligands in this aspect is available. Several previous studies reported on the significant differences between sexes, supported by the studies of genetics, epigenetics, immunology cellular physiology, and neurosciences. Sex differences in immunity have been reported many years ago but the etiology is not yet exactly understood [77,78,79,80,81].

## 3. PPAR-α and Neurosteroids in Preclinical and Clinical Therapeutic Approaches of Neurodegenerative/Neuropsychiatric Disorders

The role of PPARs as a promising target in therapy was previously described in the context of cardiovascular diseases [82,83,84,85,86]. Moreover, it was found that PPARs play an important role in chronic metabolic diseases such as T2 diabetes (DMT2), obesity, and hyperlipidemia [87,88,89,90,91]. The molecular mechanisms of PPAR-α action and its widely used agonist, fibrates, in the regulation of lipid metabolism and the refurbishment of the immune system in different pathological conditions, were previously demonstrated [16,92,93,94]. Data from clinical trials on the application of PPAR-α ligands, including fibrates, indicate that fenofibrate has anti-inflammatory and antioxidant effects [95]. This drug was approved for primary hypercholesterolemia, hypertriglyceridemia, and other dyslipidemia. It also exerts a positive effect on the blood–brain barrier (BBB). However, it is suggested that fenofibrate may exacerbate inflammatory processes and it does not exert neuroprotective effects in female animal models, as indicated in the study by Dotson et al. [96,97] and Dunn et al. [98]. It seems that this subject should be further investigated. It has been proposed that, in most cases, the positive cytoprotective effect of fenofibrate depends on tissues and their genetic arrangements. It was reported that fenofibrate has beneficial effects on brain ischemia [99], diabetic retinopathy, neuropathy, Huntington’s disease (HD), and multiple sclerosis (MS) [92,94,100,101,102]. Moreover, the benefits of fenofibrate treatment were reported in autism disorders [86]. A more precise analysis indicated that certain subgroups of patients react in a very positive way in regard to fenofibrate treatment, even much better compared to statins, which is the other class of drugs that regulate lipid metabolism. However, it is important to highlight that the FDA does not recommend the addition of fenofibrate to statin therapy. Moreover, some meta-analyses of several clinical trials indicated that fibrates may enhance the level of cystatin C and homocysteine in the blood, but this subject needs further elucidation.

The results by Dotson et al. [96,97] and Dunn et al. [98] suggest that males and females respond to PPAR-α activation differently. Dotson et al. [96] demonstrated that the PPAR-α agonist, fenofibrate, significantly improves stroke outcomes and affects inflammation in male mice but it has no effect in females. Moreover, it was found that the level of PPAR-α expression in female brains is lower compared to male brains. These data could be very important because they may indicate different responses of males and females on drugs, which historically used to be immensely neglected in therapeutic strategies. Up until now, no information has been included on this gender disparity in the clinical application of compounds containing fenofibrate, such as Lipanthyl, clofibrate, and bezafibrate. Different effects between genders could possibly be connected with the neurosteroid sex differences reported by Raciti et al. [103] and with their different levels and actions in men and women. It was reported that declining testosterone levels have been associated with an increased risk for AD and cognition alteration [104,105,106,107]. Testosterone is indicated to be neuroprotective in men and women [108,109,110]. However, hormonal therapy data demonstrated the negative effects of long-term treatment with estrogen in older post-menopausal women who are at risk of AD.

In the search for the neuroprotective effects of compounds related to PPARs, it was found that the cooperation of PPAR-α and PPAR-γ could be more efficient compared to PPAR-α alone. This could allow for the inhibition of macrophage and microglia activation, therefore preventing the entry of inflammatory cells into the CNS. The activation of these receptors may protect against the exacerbation of inflammation processes leading to neurodegeneration and neuronal death [111,112]. The study by Fuenzalida et al. [113] demonstrated that PPAR-γ increases the expression of Bcl2 antiapoptotic proteins and enhances mitochondrial function, antioxidative processes, and survival in neurons.

Several synthetic PPAR-α ligands are used in clinical treatment, as described by Bougarne et al. [16] and Sagheddu et al. [114], including clofibrate [115,116,117] and the above-mentioned fenofibrate [117,118,119], significantly influencing the lipoprotein profile in plasma. Moreover, other compounds, such as bezafibrate and gemfibrozil, decrease TG levels and are successfully used for dyslipidemia and diabetic patients [119,120]. Many novel synthetic compounds, such as WY14643, GW9578, GW6471, and GW7647, are in preclinical investigation [121,122,123]. In the study of WY14643, despite the observed positive effects, many negative results were reported. This compound induces tumorigenesis, affects liver function, and induces its enlargement to relieve neuropathic pain. Titus et al. [124] described the latest results of preclinical and clinical studies using different types of PPAR agonists to treat neuroinflammation in AD, PD, MS, cerebral ischemia, and HIV-associated neurocognitive disorders. The neuroprotective effects of gemfibrozil and bezafibrate in AD animal models were described by Chandra and Pahan [125]. Recently, Teo et al. [9] reported that oral PPAR-α agonists enhanced corneal nerve regeneration in patients with T2DM. Previously, Matlock et al. [126] demonstrated the pathogenic role of PPAR-α downregulation in corneal nerve degeneration in diabetes. He et al. [127] indicated that the PPAR-α agonist, fenofibrate, suppressed the formation of ocular surface squamous metaplasia. Sarahian et al. [128] revealed the anticonvulsive and neuroprotective effects of fenofibrate in pentylenetetrazole (PTZ)-induced-kindling seizures in mice. Moreover, fenofibrate protected the neurovascular unit and ameliorated plasma corticosterone levels in the PTZ group of mice.

After many years of fenofibrate application in cardiovascular disorders and in type 2 diabetes mellitus (T2DM), it is time to consider the application of PPARα agonist(s) in AD and other neurodegenerative disorders, particularly in the cases of patients with altered lipid metabolism. Luo et al. [50] reported that PPAR-α activation in the AD mice model (APP-PsenSEN1E9) decreased amyloid beta pathology in the hippocampus and brain cortex through autophagy regulation. Moreover, the PPAR-α agonist reduced anxiety symptoms and memory alterations in AD mice. Treatment with gemfibrozil and WY14643 enhanced autophagosome biogenesis and exerted a positive effect on the clearance of Aβ.

The following study, which was carried out in a murine AD APP/PS1 mice model, showed that synthetic PPAR-α ligands, such as GW7647, decreased lipid peroxidation and inflammation, reduced Aβ deposits, and improved cognition [129]. Moreover, in vitro experiments carried out on cells in culture (APPsw/SH-SY5Y) treated with toxic Aβ peptides demonstrated that this PPAR-α agonist enhanced the transcription of glutathione peroxidase isoform 4 (GPx4) and decreased iron transport. Recent data by Żulińska et al. [20] indicated that the synthetic PPAR-α agonist, GW7647, activates the transcription of gene-encoded proteins engaged in mitochondrial biogenesis. These include genes such as PGC-1α, NRF2, and TFAM in female AD Tg mice with the “London” mutation in APP. Concomitantly, Jamwal et al. [130] highlighted the crucial role of NRF2, uncoupling protein 2 (UCP2), and paraoxonase-2 (PON2) in PGC1-α-related mitochondrial biogenesis. Moreover, Jamwal et al. [130] proposed that PGC-1α-NRF2 signaling could be an encouraging target in a neuroprotective strategy in AD. Our experimental data using GW7647 also lead to the same conclusion and to the suggestion that the activation of the PPAR-α/PGC-1α/NRF2/TFAM pathway and mtDNA biosynthesis at an early stage of AD could be the most relevant way to delay pathology [20]. In addition, several studies focused on the role of PPAR-α ligands in the regulation of APP/Aβ metabolism by α, β, and γ secretases. In a recent study, Garcia–Gonzalez et al. [131] highlighted the emerging alternative role of membrane-type matrix metalloproteinases in APP metabolism and AD pathogenesis. These enzymes are suggested to play a significant role at the crossroads of amyloidogenesis, inflammation, and synaptic dysfunction. A previous study by Corbett et al. [132] indicated that the proteinase Adam 10 promotor contains the PPAR-α response element. Additionally, their study indicated that knockdown of PPAR-α exclusively (but not PPAR-β/δ or PPAR-γ) has a significant effect on APP metabolism. The following data demonstrate that gemfibrozil, a PPAR-α agonist through the activation of molecular events by the promotor of ADAM10, shifts APP degradation toward the alpha-secretase pathway and concomitantly decreases Aβ production by the amyloidogenic pathway, mediated by β secretase (BACE1). Moreover, in AD mouse models, gemfibrozil lowered amyloid beta plaque and improved memory [125]. However, clinical studies using PPAR ligands in AD are very poor. It is expected that in the near future, the novel promising ligands/modulators of PPARs will be available for clinical trials. Several studies using PPAR ligands are being carried out in preclinical experimental models of Parkinson’s disease (PD) as well as in clinical trials of this neurodegenerative disease and other synucleinopathy and brain ischemic pathologies [99,133]. Recently, Pérez-Segura et al. [134] summarized data from recent decades on PPARs and their protective effect in alpha synucleinopathies, including PD, dementia with Lewy bodies, multiple system atrophy (MSA), and neuroaxonal dystrophies. All these diseases are characterized by significant changes in the conformation, oligomerization, and aggregation of alpha-synuclein, a presynaptic cytosolic protein.

PD is evoked by the loss of dopaminergic neurons in substantia nigra pars compacta (SNpc), progressive alterations, and the deficiency of dopaminergic transmission in the nigrostriatal pathway. Barbiero et al. [133] reported that PPAR-α agonists might offer promising neuroprotective effects in PD. However, their study was carried out using a preclinical animal model, similar to many others published previously. It was reported that the PPAR-α ligand, fenofibrate, exerts neuroprotection in rotenone-evoked PD in male rats. Fenofibrate protected against dopaminergic neuronal cell death in the SNpc, attenuated α-synuclein aggregation, and reduced depression-like behavior and memory impairment. The study by Lee et al. [73] demonstrated the neuroprotective effects of the PPAR-α/γ dual agonist MHY908 in a 1-methyl-4-phenyl-1,2,3,6-tetrahydropyridine (MPTP)-evoked Parkinson’s experimental model. This compound decreases dopaminergic neuron loss, motor alteration, and inflammatory processes. Previous preclinical studies highlighted the therapeutic effects of PPAR-γ agonists in PD but all clinical trials were negative. Nevertheless, the agonist of the rosiglitazone receptor exerted positive therapeutic effects by improving cognitive function in a preclinical AD study and in treating patients with AD [135,136,137,138]. Unfortunately, the positive effects of other drugs (such as Pioglitazone) in AD patients are controversial. The data presented by Geldmacher et al. [139] and Galimberti et al. [140] demonstrated a lack of efficacy in the clinical treatment of this neurodegenerative disease. The study by Chandra and Pahan [125] showed that gemfibrozil (an FDA-approved drug for hyperlipidemia) through PPAR-α decreased amyloid plaque and improved memory in a mouse model of AD (in 5xFAD).

It seems that agonists of PPAR-γ and PPAR-α may offer promising effects in depression as adjunctive treatment in PD/AD and schizophrenia [141,142]. It was found that PPAR-α knockdown mice exhibited a schizophrenia-relevant phenotype that included behavioral deficits and impaired synaptogenesis in the cerebral cortex. PPAR-α regulates the expression of genes engaged in synaptogenesis. Several studies proposed that the mechanism underlying schizophrenia pathogenesis involves PPAR-α-regulated transcription of several genes related to synaptogenesis and synaptic function [143,144,145]. PPAR-α and other members of the ligand-activated nuclear receptors are implicated in several neurodegenerative and neurodevelopmental disorders and systemic human pathologies, including arteriosclerosis, diabetes type 2, and inflammation.

It is indicated that PPARs play a significant role in neuroinflammation and the brain–gut microbiota axis, as well as their anti-inflammatory properties [146]. PPAR-α is highly abundant in the gastrointestinal (GI) tract. The close link between PPARs, bile acids, the brain–gut microbiota axis, and immune homeostasis has been recently proposed [147,148]. PPAR-α, as mentioned above, is engaged in the regulation of gene-coding sulfotransferases, which are responsible for the sulfation of bile acid (BA). The presence of BA and its receptors in the brain exert a significant effect on brain function and inflammation. Moreover, the brain–gut microbiota axis may affect the course and progression of AD/PD as well as other brain disorders. Inflammatory signaling occurs across the gut–brain axis in both directions (gut to brain and brain to gut). A better understanding of this bidirectional axis and neuroimmune relationship in AD, as well as several other neurological/neuropsychiatric diseases, should help improve therapeutic strategies.

## 4. Conclusions

In this review, we demonstrated the latest view on the role of the interaction between the nuclear receptor PPAR-α and neurosteroid synthesis in the brain during aging and neurodegenerative/neuropsychiatric disorders. Moreover, differences between males and females in the expression of genes coding PPARs, steroidogenic enzymes, and levels of circulating steroids are described in relation to a therapeutic strategy targeting PPAR receptors. Up until now, it has been difficult to evaluate the concentration of neurosteroids in different parts of the human brain in a gender-specific manner in physiological and pathological conditions. It should be highlighted that gene expression for PPAR-α, cholesterol/neurosteroid enzymes, and signaling pathways could be species-specific, and the data from different experimental models ought to be considered carefully.

Recently, several novel pharmacologically active compound agonists, PPAR-α ligands, and neurosteroids have been investigated, and some were introduced to the treatment of depression, which should significantly improve the therapeutic strategies of neuropsychiatric disorders. However, many side effects were also reported; this aspect should be very carefully considered during their application. Moreover, the signaling pathways evoked by PPAR-α and its interaction with other receptors from this family, such as PPAR-γ, may enhance the chances of improving neuroprotection in AD and other neurodegenerative/neuropsychiatric disorders. Additionally, the neuroprotective effect of neurosteroids via GABAA receptor-mediated signaling should be promising in the treatment of AD/PD, peripheral neuropathies, and neuropathic pain. Neuroactive steroids, apart from the activation of specific steroid membrane receptors through modulation of GABAA and GABAB, exert additional effects on NMDA and 5-HT3. Neurosteroids synthesized in the brain, as well as circulating steroids synthesized in peripheral organs, which readily cross the blood–brain barrier (BBA), significantly affect neuroinflammation, which is a crucial component of AD, PD, MS, and other neuropsychiatric disorders, such as schizophrenia or bipolar disorders. However, it should be noted that inflammation might influence neurosteroid levels and actions. Over the last 5 years, the FDA approved three novel compounds, including neurosteroids for the treatment of depression. To summarize, it can be expected that in the near future, novel synthetic PPAR-α ligand receptors, as well as novel synthetic neurosteroid analogs, enantiomers, and derivatives, will be able to improve the treatment of neurodegenerative/neuropsychiatric disorders.

## Data Availability

Not applicable.
